# Tricarbon­yl[*N*,*N*′,*N*′′-tris­(2,6-diisopropyl­phen­yl)guanidine]molybdenum(0)

**DOI:** 10.1107/S1600536811021441

**Published:** 2011-06-11

**Authors:** René T. Boeré, Jason D. Masuda

**Affiliations:** a Department of Chemistry and Biochemistry, University of Lethbridge, Lethbridge, AB, Canada T1K 3M4; b The Maritime Centre for Green Chemistry and the Department of Chemistry, Saint Mary’s University, Halifax, NS, Canada B3H 3C3

## Abstract

In the title compound, [Mo(C_37_H_53_N_3_)(CO)_3_], the Mo atom to ring-centroid distance in the η^6^-coordinated tricarbonyl­molybdenum group is 1.958 (1) Å. The three C O groups are pseudo-octa­hedrally disposed with C—Mo—C angles ranging from 80.7 (1) to 87.4 (1)°. The two uncoordinated 2,6-diisopropyl­phenyl-substituted benzene rings form dihedral angles of 75.96 (8) and 78.01 (9)° with the mean plane of the guanidine group. The coordinated benzene ring is in a slight sofa conformation with the *N*-substituted C atom and the bonded N atom dispaced by 0.090 (3) and 0.458 (4) Å, respectively, from the mean plane of the remaining ring atoms. In the crystal, despite there being two N—H donor groups, no conventional hydrogen bonds are present. This may be because of the steric effects of the bulky diisopropyl­phenyl groups.

## Related literature

For the structure of the parent guanidine ligand, see: Boeré, Boeré *et al.* (2000 ▶). For a series of related guanidines with varying conformational isomers, see: Gopi *et al.* (2010 ▶). For applications of this same ligand with cobalt(II) for catalysis, see: Eichman *et al.* (2011 ▶). For the use of a closely related ligand synthesized in an analogous manner, see: Brazeau *et al.* (2011 ▶). For a comprehensive review of the coordination chemistry of neutral guanidines, see: Coles (2006 ▶). For related amidine complexes in which Mo(CO)_3_ is coordinated in a very similar manner, see; Boeré, Klassen & Wolmershäuser (1998 ▶, 2000 ▶). For thermal motion of carbonyl group oxygen atoms, see: Braga & Koetzle (1988 ▶)
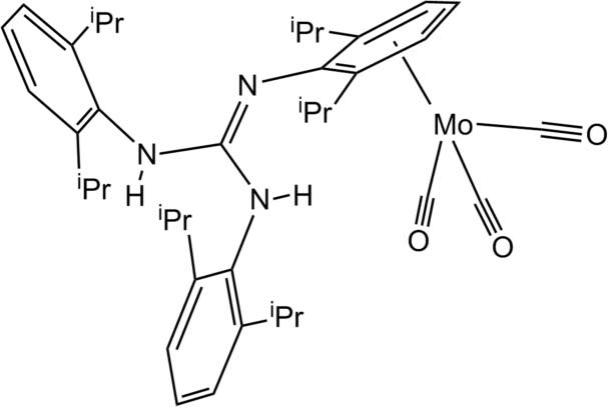


## Experimental

### 

#### Crystal data

[Mo(C_37_H_53_N_3_)(CO)_3_]
*M* *_r_* = 719.79
Triclinic, 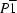

*a* = 10.6525 (12) Å
*b* = 11.7642 (14) Å
*c* = 16.5482 (19) Å
α = 89.128 (1)°
β = 78.713 (1)°
γ = 67.240 (1)°
*V* = 1871.1 (4) Å^3^
*Z* = 2
Mo *K*α radiation
μ = 0.39 mm^−1^
*T* = 173 K
0.29 × 0.12 × 0.11 mm

#### Data collection

Bruker APEXII CCD area-detector diffractometer
Absorption correction: multi-scan (*SADABS*; Bruker, 2006 ▶) *T*_min_ = 0.705, *T*_max_ = 0.746
27034 measured reflections
8399 independent reflections
6634 reflections with *I* > 2σ(*I*)
*R*_int_ = 0.042

#### Refinement

*R*[*F* ^2^ > 2σ(*F* ^2^)] = 0.038
*wR*(*F* ^2^) = 0.115
*S* = 1.05
8399 reflections
442 parameters
2 restraints
H atoms treated by a mixture of independent and constrained refinement
Δρ_max_ = 1.12 e Å^−3^
Δρ_min_ = −0.59 e Å^−3^

### 

Data collection: *APEX2* (Bruker, 2006 ▶); cell refinement: *SAINT-Plus* (Bruker, 2006 ▶); data reduction: *SAINT-Plus*; program(s) used to solve structure: *SHELXS97* (Sheldrick, 2008 ▶); program(s) used to refine structure: *SHELXTL* (Sheldrick, 2008 ▶); molecular graphics: *Mercury* (Macrae *et al.*, 2006 ▶); software used to prepare material for publication: *publCIF* (Westrip, 2010 ▶).

## Supplementary Material

Crystal structure: contains datablock(s) I, global. DOI: 10.1107/S1600536811021441/lh5261sup1.cif

Structure factors: contains datablock(s) I. DOI: 10.1107/S1600536811021441/lh5261Isup2.hkl

Additional supplementary materials:  crystallographic information; 3D view; checkCIF report

## Figures and Tables

**Table 1:** Comparison of inter­atomic distances and angles (Å, °) of (I) with free guanidine, (II)

	C1—N1	C1—N2	C1—N3	N1—C1—N2	N2—C1—N3	N3—C1—N1
(I)	1.287 (3)	1.361 (3)	1.374 (3)	125.0 (2)	115.57 (19)	119.42 (19)
(II)	1.316 (2)	1.348 (2)	1.357 (2)	121.99 (13)	118.47 (14)	119.52 (13)

**Fig. 1.**
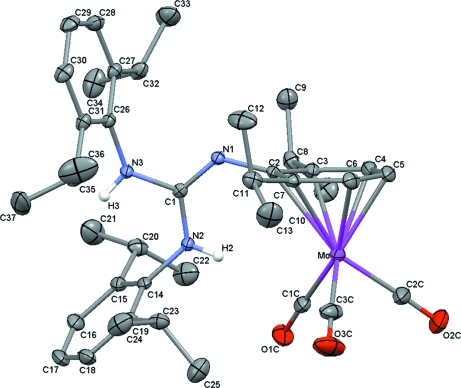
The molecular structure of (I) shown with 30% probability ellipsoids. H atoms bonded to C atoms are not shown.

## supplementary crystallographic information

### Comment

The molecular stucture of the title compound, (I), is shown in Figure 1. The X-ray crystal structure of *N*,*N*',*N*"-tris(2,6-diisopropylphenyl)guanidine (II) was reported by Boeré, Boeré *et al.* (2000) with the three aryl groups in the same *syn*-*anti* conformation (Gopi *et al.*, 2010) as found in compound (I). Table 1 presents the selected geometric data for compounds (I) and (II). Coles (2006) has comprehensively reviewed the application of neutral amidines and guanidines as coordination ligands. Recently, a cobalt(II) complex of the title ligand has been used as a catalyst in the synthesis of polysubstituted arenes *via* the regioselective cyclotrimerization of alkynes (Eichman *et al.*, 2011). Also, deprotonated *N*,*N*',*N*"-aryl guandines have been reported to stabilize low-coordinate As(III) cations (Brazeau *et al.*, 2011).

The title compound has an η^6^–coordinated tricarbonylmolybdenum group with a Mo to ring-centroid distance of 1.958 (1)Å. The three C≡O groups are pseudooctahedraly disposed with C–Mo–C angles ranging from 80.7 (1) to 87.4 (1)°. The three 2,6-diisopropylphenyl rings have normals that are disposed at 79.78 (8)° (C3-C7), 75.96 (8)° (C14-C19) and 78.01 (9)° (C26-C31) to the guanidine plane defined by C1, N1-N3. The ring coordinated by Mo(CO)_3_ is bent back from the core such that C2 is located 0.090 (3) and N1 0.458 (4)Å from the plane defined by C3-C7. In the crystal, despite there being two N—H donor groups, no conventional hydrogen bonds are present. This is possibly due to the steric effects of the bulky diisopropylphenyl groups. The orientation of the Mo(CO)_3_ unit and its geometric parameters are found to be very similar in compound (I) and in closely comparable tricarbonylmolybdenum complexes of structurally similar amidines (Boeré, Klassen & Wolmershäuser,  1998, 2000). The observed outward bending of the coordinated aryl ring suggests that some steric effects operate between the amidine/guanidine groups and the Mo(CO)_3_ units.

### Experimental

The compound was prepared by a thermal reaction between the neural guanidine ligand and Mo(CO)_6_ as described in Boeré, Boeré *et al.* (2000). Full characterization by elemental analysis, NMR, mass spectrometry and infra-red spectroscopy are provided there.

### Refinement

Hydrogen atoms attached to carbon were refined using a riding model with temperature factors of 1.2 (CH) or 1.5 (CH_3_) × the equivalent isotropic values of the attached atoms. H2 and H3 attached to nitrogen were positionally refined using distance restraints of 0.88 Å and temperature factors 1.2 × the equivalent isotropic values of N2 and N3. Two reflections have unusually large deviations from the weighted errors of their intensities; no obvious cause could be determined for this effect. The isopropyl methyl groups are found to librate more than other carbon atoms but this effect is commonly observed in 2,6-diisopropylphenyl compounds. A rotational disorder model for isopropyl groups was judged to be unwarranted. Similarly, the carbonyl group oxygen atoms display considerable thermal motion, but this is also a well known behaviour, see Braga & Koetzle (1988).

### Crystal data

**Table:**

[Mo(C_37_H_53_N_3_)(CO)_3_]	*Z* = 2
*M**_r_* = 719.79	*F*(000) = 760
Triclinic, *P*1	*D*_x_ = 1.278 Mg m^−^^3^
Hall symbol: -P 1	Melting point: 483 K
*a* = 10.6525 (12) Å	Mo *K*α radiation, λ = 0.71073 Å
*b* = 11.7642 (14) Å	Cell parameters from 8978 reflections
*c* = 16.5482 (19) Å	θ = 2.2–26.7°
α = 89.128 (1)°	µ = 0.39 mm^−^^1^
β = 78.713 (1)°	*T* = 173 K
γ = 67.240 (1)°	Block, yellow
*V* = 1871.1 (4) Å^3^	0.29 × 0.12 × 0.11 mm

### Data collection

**Table:**

Bruker APEXII CCD area-detector diffractometer	8399 independent reflections
Radiation source: X-ray	6634 reflections with *I* > 2σ(*I*)
graphite	*R*_int_ = 0.042
Detector resolution: 0.015 pixels mm^-1^	θ_max_ = 27.4°, θ_min_ = 1.9°
φ and ω scans	*h* = −13→13
Absorption correction: multi-scan (*SADABS*; Bruker, 2006)	*k* = −15→15
*T*_min_ = 0.705, *T*_max_ = 0.746	*l* = −21→21
27034 measured reflections	

### Refinement

**Table:**

Refinement on *F*^2^	Primary atom site location: structure-invariant direct methods
Least-squares matrix: full	Secondary atom site location: difference Fourier map
*R*[*F*^2^ > 2σ(*F*^2^)] = 0.038	Hydrogen site location: inferred from neighbouring sites
*wR*(*F*^2^) = 0.115	H atoms treated by a mixture of independent and constrained refinement
*S* = 1.05	*w* = 1/[σ^2^(*F*_o_^2^) + (0.0637*P*)^2^] where *P* = (*F*_o_^2^ + 2*F*_c_^2^)/3
8399 reflections	(Δ/σ)_max_ = 0.001
442 parameters	Δρ_max_ = 1.12 e Å^−^^3^
2 restraints	Δρ_min_ = −0.59 e Å^−^^3^
0 constraints	

### Special details

**Table:**

Experimental. A crystal coated in Paratone (TM) oil was mounted on the end of a thin glass capillary and cooled in the gas stream of the diffractometer Kryoflex low temperature device.
Geometry. All e.s.d.'s (except the e.s.d. in the dihedral angle between two l.s. planes) are estimated using the full covariance matrix. The cell e.s.d.'s are taken into account individually in the estimation of e.s.d.'s in distances, angles and torsion angles; correlations between e.s.d.'s in cell parameters are only used when they are defined by crystal symmetry. An approximate (isotropic) treatment of cell e.s.d.'s is used for estimating e.s.d.'s involving l.s. planes.
Refinement. Refinement of *F*^2^ against ALL reflections. The weighted *R*-factor *wR* and goodness of fit *S* are based on *F*^2^, conventional *R*-factors *R* are based on *F*, with *F* set to zero for negative *F*^2^. The threshold expression of *F*^2^ > σ(*F*^2^) is used only for calculating *R*-factors(gt) *etc*. and is not relevant to the choice of reflections for refinement. *R*-factors based on *F*^2^ are statistically about twice as large as those based on *F*, and *R*- factors based on ALL data will be even larger. In the final cycle of LS refinement an unusually large residual peak of 1.13 e^-^/A^3^ was located about midway between carbonyl C1C and C2C. Though this might indicated positional disorder of the tripodal (CO)_3_ group, no similar peaks were found between the remaining C1C - C3C and C3C - C2C carbonyl groups. Finally, the model includes two NH groups that are potential H-bond donors. However H-bonding is not observed, probably due to steric constraints.

### Fractional atomic coordinates and isotropic or equivalent isotropic displacement parameters (Å^2^)

**Table:**

	*x*	*y*	*z*	*U*_iso_*/*U*_eq_	
Mo	0.71984 (2)	0.914384 (19)	0.598865 (13)	0.03364 (9)	
O1C	0.6521 (2)	1.06506 (19)	0.76505 (13)	0.0555 (6)	
O2C	0.6473 (3)	1.1793 (2)	0.54066 (17)	0.0972 (11)	
O3C	0.4025 (2)	0.9815 (3)	0.61834 (16)	0.0787 (8)	
C1C	0.6772 (3)	1.0059 (2)	0.70321 (17)	0.0374 (6)	
C2C	0.6744 (4)	1.0793 (3)	0.56057 (19)	0.0597 (10)	
C3C	0.5213 (3)	0.9546 (3)	0.61212 (18)	0.0513 (8)	
N1	0.91757 (19)	0.62265 (16)	0.68815 (11)	0.0214 (4)	
N2	0.7736 (2)	0.76440 (17)	0.79907 (12)	0.0216 (4)	
H2	0.743 (2)	0.812 (2)	0.7652 (13)	0.026*	
N3	0.9044 (2)	0.56192 (16)	0.82112 (11)	0.0214 (4)	
H3	0.852 (2)	0.580 (2)	0.8656 (12)	0.026*	
C1	0.8647 (2)	0.65043 (19)	0.76564 (13)	0.0184 (4)	
C2	0.8854 (2)	0.7025 (2)	0.62515 (13)	0.0227 (5)	
C3	0.9571 (2)	0.7836 (2)	0.60211 (14)	0.0256 (5)	
C4	0.9517 (3)	0.8349 (2)	0.52481 (15)	0.0321 (6)	
H4	1.0042	0.8834	0.5070	0.039*	
C5	0.8699 (3)	0.8155 (2)	0.47345 (15)	0.0366 (6)	
H5	0.8686	0.8498	0.4211	0.044*	
C6	0.7917 (3)	0.7467 (2)	0.49918 (15)	0.0332 (6)	
H6	0.7335	0.7374	0.4653	0.040*	
C7	0.7965 (2)	0.6890 (2)	0.57589 (14)	0.0259 (5)	
C8	1.0516 (3)	0.7977 (2)	0.65510 (16)	0.0327 (6)	
H8	1.0155	0.7833	0.7131	0.039*	
C9	1.1982 (3)	0.6989 (3)	0.62729 (19)	0.0457 (7)	
H9A	1.1940	0.6171	0.6269	0.069*	
H9B	1.2563	0.7025	0.6655	0.069*	
H9C	1.2384	0.7135	0.5716	0.069*	
C10	1.0555 (4)	0.9255 (3)	0.6556 (2)	0.0520 (8)	
H10A	1.1001	0.9387	0.6006	0.078*	
H10B	1.1087	0.9321	0.6961	0.078*	
H10C	0.9604	0.9882	0.6703	0.078*	
C11	0.7265 (3)	0.5997 (2)	0.59820 (16)	0.0323 (6)	
H11	0.7080	0.5976	0.6597	0.039*	
C12	0.8290 (3)	0.4709 (3)	0.5621 (2)	0.0552 (9)	
H12A	0.8534	0.4713	0.5019	0.083*	
H12B	0.7860	0.4112	0.5766	0.083*	
H12C	0.9133	0.4475	0.5848	0.083*	
C13	0.5881 (3)	0.6336 (3)	0.5711 (2)	0.0605 (9)	
H13A	0.5228	0.7149	0.5968	0.091*	
H13B	0.5497	0.5716	0.5883	0.091*	
H13C	0.6030	0.6362	0.5109	0.091*	
C14	0.6968 (2)	0.79018 (19)	0.88280 (14)	0.0233 (5)	
C15	0.7616 (3)	0.8093 (2)	0.94427 (15)	0.0275 (5)	
C16	0.6854 (3)	0.8331 (2)	1.02566 (16)	0.0375 (6)	
H16	0.7268	0.8451	1.0689	0.045*	
C17	0.5508 (3)	0.8392 (3)	1.04368 (17)	0.0443 (7)	
H17	0.5006	0.8552	1.0993	0.053*	
C18	0.4895 (3)	0.8225 (2)	0.98302 (17)	0.0381 (6)	
H18	0.3962	0.8286	0.9969	0.046*	
C19	0.5602 (2)	0.7966 (2)	0.90043 (15)	0.0280 (5)	
C20	0.9061 (3)	0.8110 (2)	0.92375 (16)	0.0349 (6)	
H20	0.9601	0.7517	0.8747	0.042*	
C21	0.9865 (4)	0.7713 (4)	0.9927 (2)	0.0661 (10)	
H21A	0.9423	0.8333	1.0395	0.099*	
H21B	1.0825	0.7635	0.9726	0.099*	
H21C	0.9865	0.6915	1.0106	0.099*	
C22	0.8981 (3)	0.9385 (3)	0.8993 (2)	0.0570 (9)	
H22A	0.8514	0.9616	0.8526	0.086*	
H22B	0.9923	0.9371	0.8835	0.086*	
H22C	0.8455	0.9990	0.9462	0.086*	
C23	0.4864 (3)	0.7839 (2)	0.83388 (17)	0.0339 (6)	
H23	0.5582	0.7473	0.7823	0.041*	
C24	0.4066 (3)	0.6998 (3)	0.8554 (2)	0.0529 (8)	
H24A	0.4683	0.6210	0.8722	0.079*	
H24B	0.3738	0.6846	0.8069	0.079*	
H24C	0.3268	0.7399	0.9007	0.079*	
C25	0.3861 (3)	0.9100 (3)	0.8160 (2)	0.0516 (8)	
H25A	0.3144	0.9476	0.8658	0.077*	
H25B	0.3422	0.9005	0.7712	0.077*	
H25C	0.4372	0.9631	0.7996	0.077*	
C26	0.9760 (2)	0.43247 (19)	0.79664 (13)	0.0217 (5)	
C27	1.1210 (2)	0.3814 (2)	0.77456 (14)	0.0248 (5)	
C28	1.1875 (3)	0.2556 (2)	0.75388 (16)	0.0323 (6)	
H28	1.2861	0.2192	0.7379	0.039*	
C29	1.1121 (3)	0.1821 (2)	0.75617 (16)	0.0357 (6)	
H29	1.1592	0.0959	0.7421	0.043*	
C30	0.9694 (3)	0.2336 (2)	0.77869 (16)	0.0329 (6)	
H30	0.9189	0.1822	0.7801	0.039*	
C31	0.8980 (2)	0.3592 (2)	0.79933 (15)	0.0257 (5)	
C32	1.2042 (3)	0.4605 (2)	0.77695 (16)	0.0323 (6)	
H32	1.1474	0.5456	0.7631	0.039*	
C33	1.3426 (3)	0.4132 (3)	0.7144 (2)	0.0553 (9)	
H33A	1.4044	0.3336	0.7301	0.083*	
H33B	1.3863	0.4729	0.7136	0.083*	
H33C	1.3259	0.4029	0.6593	0.083*	
C34	1.2277 (4)	0.4691 (3)	0.8638 (2)	0.0538 (9)	
H34A	1.1381	0.4986	0.9030	0.081*	
H34B	1.2729	0.5270	0.8666	0.081*	
H34C	1.2874	0.3874	0.8778	0.081*	
C35	0.7412 (3)	0.4149 (2)	0.82715 (18)	0.0363 (6)	
H35	0.7079	0.5030	0.8126	0.044*	
C36	0.6700 (4)	0.3517 (4)	0.7839 (3)	0.0876 (15)	
H36A	0.7077	0.3429	0.7243	0.131*	
H36B	0.5698	0.4015	0.7947	0.131*	
H36C	0.6868	0.2698	0.8047	0.131*	
C37	0.7001 (3)	0.4148 (4)	0.9208 (2)	0.0722 (12)	
H37A	0.7309	0.3295	0.9371	0.108*	
H37B	0.5988	0.4557	0.9382	0.108*	
H37C	0.7441	0.4593	0.9471	0.108*	

### Atomic displacement parameters (Å^2^)

**Table:**

	*U*^11^	*U*^22^	*U*^33^	*U*^12^	*U*^13^	*U*^23^
Mo	0.03955 (15)	0.02627 (13)	0.02197 (13)	0.00019 (9)	−0.00416 (9)	0.00151 (8)
O1C	0.0845 (17)	0.0362 (11)	0.0350 (12)	−0.0190 (11)	0.0031 (11)	−0.0101 (9)
O2C	0.135 (3)	0.0408 (14)	0.0652 (17)	0.0043 (15)	0.0105 (17)	0.0229 (12)
O3C	0.0403 (14)	0.104 (2)	0.0611 (16)	0.0089 (13)	−0.0167 (12)	−0.0177 (15)
C1C	0.0464 (16)	0.0236 (12)	0.0330 (15)	−0.0066 (12)	−0.0023 (12)	0.0044 (11)
C2C	0.077 (2)	0.0351 (16)	0.0340 (17)	0.0058 (15)	0.0049 (16)	0.0065 (13)
C3C	0.0461 (19)	0.0531 (19)	0.0321 (16)	0.0067 (15)	−0.0106 (13)	−0.0108 (13)
N1	0.0253 (10)	0.0186 (9)	0.0187 (9)	−0.0064 (8)	−0.0051 (8)	−0.0003 (7)
N2	0.0247 (10)	0.0186 (9)	0.0182 (10)	−0.0054 (8)	−0.0038 (8)	0.0027 (7)
N3	0.0263 (10)	0.0178 (9)	0.0178 (10)	−0.0067 (8)	−0.0036 (8)	0.0004 (7)
C1	0.0177 (10)	0.0188 (10)	0.0224 (11)	−0.0099 (9)	−0.0065 (9)	0.0012 (8)
C2	0.0253 (12)	0.0197 (11)	0.0162 (11)	−0.0028 (9)	−0.0008 (9)	−0.0029 (8)
C3	0.0306 (13)	0.0212 (11)	0.0212 (12)	−0.0079 (10)	−0.0013 (10)	−0.0013 (9)
C4	0.0409 (15)	0.0233 (12)	0.0265 (13)	−0.0102 (11)	0.0010 (11)	0.0014 (10)
C5	0.0473 (16)	0.0310 (13)	0.0180 (12)	−0.0029 (12)	−0.0023 (11)	0.0010 (10)
C6	0.0370 (14)	0.0366 (14)	0.0196 (12)	−0.0056 (11)	−0.0093 (10)	−0.0035 (10)
C7	0.0252 (12)	0.0242 (11)	0.0226 (12)	−0.0038 (10)	−0.0037 (9)	−0.0048 (9)
C8	0.0403 (15)	0.0355 (14)	0.0280 (13)	−0.0227 (12)	−0.0038 (11)	0.0012 (11)
C9	0.0423 (17)	0.0530 (18)	0.0468 (18)	−0.0219 (14)	−0.0140 (14)	0.0087 (14)
C10	0.064 (2)	0.0426 (17)	0.062 (2)	−0.0329 (16)	−0.0135 (17)	−0.0022 (15)
C11	0.0312 (13)	0.0356 (14)	0.0321 (14)	−0.0142 (11)	−0.0079 (11)	−0.0038 (11)
C12	0.0515 (19)	0.0382 (16)	0.074 (2)	−0.0225 (15)	0.0038 (17)	−0.0221 (15)
C13	0.0406 (18)	0.076 (2)	0.076 (2)	−0.0293 (18)	−0.0241 (17)	0.013 (2)
C14	0.0263 (12)	0.0153 (10)	0.0212 (11)	−0.0031 (9)	0.0003 (9)	0.0005 (8)
C15	0.0308 (13)	0.0226 (11)	0.0239 (12)	−0.0056 (10)	−0.0038 (10)	−0.0006 (9)
C16	0.0478 (17)	0.0331 (14)	0.0231 (13)	−0.0077 (12)	−0.0044 (12)	−0.0040 (10)
C17	0.0516 (18)	0.0375 (15)	0.0249 (14)	−0.0064 (13)	0.0116 (13)	−0.0014 (11)
C18	0.0321 (14)	0.0330 (14)	0.0386 (16)	−0.0091 (11)	0.0095 (12)	−0.0008 (11)
C19	0.0273 (12)	0.0193 (11)	0.0319 (13)	−0.0061 (10)	0.0000 (10)	0.0004 (9)
C20	0.0352 (14)	0.0382 (14)	0.0282 (14)	−0.0103 (12)	−0.0075 (11)	−0.0074 (11)
C21	0.051 (2)	0.095 (3)	0.049 (2)	−0.019 (2)	−0.0227 (17)	0.0062 (19)
C22	0.0516 (19)	0.0476 (19)	0.077 (2)	−0.0270 (16)	−0.0093 (17)	0.0006 (17)
C23	0.0262 (13)	0.0328 (13)	0.0401 (15)	−0.0109 (11)	−0.0023 (11)	−0.0039 (11)
C24	0.0420 (17)	0.0404 (17)	0.083 (2)	−0.0205 (14)	−0.0194 (17)	0.0075 (16)
C25	0.059 (2)	0.0410 (17)	0.062 (2)	−0.0202 (15)	−0.0273 (17)	0.0126 (15)
C26	0.0273 (12)	0.0182 (10)	0.0198 (11)	−0.0075 (9)	−0.0082 (9)	0.0023 (8)
C27	0.0263 (12)	0.0244 (11)	0.0248 (12)	−0.0094 (10)	−0.0093 (10)	0.0030 (9)
C28	0.0279 (13)	0.0278 (13)	0.0352 (14)	−0.0032 (10)	−0.0092 (11)	0.0003 (10)
C29	0.0437 (15)	0.0197 (12)	0.0385 (15)	−0.0050 (11)	−0.0117 (12)	−0.0040 (10)
C30	0.0419 (15)	0.0231 (12)	0.0409 (15)	−0.0162 (11)	−0.0176 (12)	0.0022 (11)
C31	0.0297 (12)	0.0237 (11)	0.0275 (13)	−0.0118 (10)	−0.0114 (10)	0.0048 (9)
C32	0.0263 (13)	0.0301 (13)	0.0433 (16)	−0.0124 (11)	−0.0112 (11)	0.0087 (11)
C33	0.0300 (15)	0.0543 (19)	0.077 (2)	−0.0161 (14)	−0.0033 (15)	0.0110 (17)
C34	0.073 (2)	0.0541 (19)	0.063 (2)	−0.0444 (18)	−0.0377 (18)	0.0156 (16)
C35	0.0300 (14)	0.0335 (14)	0.0526 (17)	−0.0170 (11)	−0.0158 (12)	0.0135 (12)
C36	0.045 (2)	0.071 (3)	0.162 (5)	−0.0293 (19)	−0.040 (3)	−0.015 (3)
C37	0.0369 (18)	0.097 (3)	0.064 (2)	−0.0136 (18)	0.0020 (16)	0.038 (2)

### Geometric parameters (Å, °)

**Table:**

Mo—C1C	1.928 (3)	C19—C23	1.513 (4)
Mo—C2C	1.938 (3)	C20—C21	1.520 (4)
O1C—C1C	1.172 (3)	C20—C22	1.522 (4)
O2C—C2C	1.156 (4)	C20—H20	1.0000
O3C—C3C	1.164 (4)	C21—H21A	0.9800
N1—C1	1.287 (3)	C21—H21B	0.9800
N1—C2	1.395 (3)	C21—H21C	0.9800
N2—C1	1.361 (3)	C22—H22A	0.9800
N2—C14	1.435 (3)	C22—H22B	0.9800
N2—H2	0.809 (16)	C22—H22C	0.9800
N3—C1	1.374 (3)	C23—C25	1.521 (4)
N3—C26	1.437 (3)	C23—C24	1.533 (4)
N3—H3	0.807 (16)	C23—H23	1.0000
C2—C7	1.417 (3)	C24—H24A	0.9800
C3—C4	1.408 (3)	C24—H24B	0.9800
C4—C5	1.408 (4)	C24—H24C	0.9800
C4—H4	0.9500	C25—H25A	0.9800
C5—C6	1.379 (4)	C25—H25B	0.9800
C5—H5	0.9500	C25—H25C	0.9800
C6—C7	1.431 (3)	C26—C27	1.395 (3)
C6—H6	0.9500	C26—C31	1.406 (3)
C8—C10	1.520 (4)	C27—C28	1.386 (3)
C8—C9	1.527 (4)	C27—C32	1.518 (3)
C8—H8	1.0000	C28—C29	1.385 (4)
C9—H9A	0.9800	C28—H28	0.9500
C9—H9B	0.9800	C29—C30	1.374 (4)
C9—H9C	0.9800	C29—H29	0.9500
C10—H10A	0.9800	C30—C31	1.387 (3)
C10—H10B	0.9800	C30—H30	0.9500
C10—H10C	0.9800	C31—C35	1.514 (3)
C11—C13	1.527 (4)	C32—C34	1.518 (4)
C11—C12	1.529 (4)	C32—C33	1.530 (4)
C11—H11	1.0000	C32—H32	1.0000
C12—H12A	0.9800	C33—H33A	0.9800
C12—H12B	0.9800	C33—H33B	0.9800
C12—H12C	0.9800	C33—H33C	0.9800
C13—H13A	0.9800	C34—H34A	0.9800
C13—H13B	0.9800	C34—H34B	0.9800
C13—H13C	0.9800	C34—H34C	0.9800
C14—C19	1.398 (3)	C35—C36	1.518 (4)
C14—C15	1.401 (3)	C35—C37	1.525 (4)
C15—C16	1.400 (3)	C35—H35	1.0000
C15—C20	1.519 (4)	C36—H36A	0.9800
C16—C17	1.379 (4)	C36—H36B	0.9800
C16—H16	0.9500	C36—H36C	0.9800
C17—C18	1.355 (4)	C37—H37A	0.9800
C17—H17	0.9500	C37—H37B	0.9800
C18—C19	1.400 (3)	C37—H37C	0.9800
C18—H18	0.9500		
			
C1C—Mo—C2C	80.69 (12)	C22—C20—H20	107.5
O1C—C1C—Mo	177.4 (2)	C20—C21—H21A	109.5
O2C—C2C—Mo	177.4 (3)	C20—C21—H21B	109.5
O3C—C3C—Mo	177.9 (3)	H21A—C21—H21B	109.5
C1—N1—C2	125.51 (19)	C20—C21—H21C	109.5
C1—N2—C14	124.59 (18)	H21A—C21—H21C	109.5
C1—N2—H2	113.7 (18)	H21B—C21—H21C	109.5
C14—N2—H2	117.9 (18)	C20—C22—H22A	109.5
C1—N3—C26	122.77 (18)	C20—C22—H22B	109.5
C1—N3—H3	112.9 (18)	H22A—C22—H22B	109.5
C26—N3—H3	116.6 (18)	C20—C22—H22C	109.5
N1—C1—N2	125.0 (2)	H22A—C22—H22C	109.5
N1—C1—N3	119.41 (19)	H22B—C22—H22C	109.5
N2—C1—N3	115.57 (19)	C19—C23—C25	110.5 (2)
N1—C2—C7	118.4 (2)	C19—C23—C24	113.4 (2)
C4—C3—C2	118.5 (2)	C25—C23—C24	108.9 (2)
C3—C4—C5	121.1 (2)	C19—C23—H23	108.0
C3—C4—H4	119.4	C25—C23—H23	108.0
C5—C4—H4	119.4	C24—C23—H23	108.0
C6—C5—C4	120.0 (2)	C23—C24—H24A	109.5
C6—C5—H5	120.0	C23—C24—H24B	109.5
C4—C5—H5	120.0	H24A—C24—H24B	109.5
C5—C6—C7	121.4 (2)	C23—C24—H24C	109.5
C5—C6—H6	119.3	H24A—C24—H24C	109.5
C7—C6—H6	119.3	H24B—C24—H24C	109.5
C2—C7—C6	118.5 (2)	C23—C25—H25A	109.5
C3—C8—C10	113.4 (2)	C23—C25—H25B	109.5
C3—C8—C9	109.8 (2)	H25A—C25—H25B	109.5
C10—C8—C9	110.4 (2)	C23—C25—H25C	109.5
C3—C8—H8	107.6	H25A—C25—H25C	109.5
C10—C8—H8	107.6	H25B—C25—H25C	109.5
C9—C8—H8	107.6	C27—C26—C31	121.6 (2)
C8—C9—H9A	109.5	C27—C26—N3	119.6 (2)
C8—C9—H9B	109.5	C31—C26—N3	118.8 (2)
H9A—C9—H9B	109.5	C28—C27—C26	118.3 (2)
C8—C9—H9C	109.5	C28—C27—C32	120.7 (2)
H9A—C9—H9C	109.5	C26—C27—C32	120.9 (2)
H9B—C9—H9C	109.5	C29—C28—C27	120.8 (2)
C8—C10—H10A	109.5	C29—C28—H28	119.6
C8—C10—H10B	109.5	C27—C28—H28	119.6
H10A—C10—H10B	109.5	C30—C29—C28	120.2 (2)
C8—C10—H10C	109.5	C30—C29—H29	119.9
H10A—C10—H10C	109.5	C28—C29—H29	119.9
H10B—C10—H10C	109.5	C29—C30—C31	121.1 (2)
C7—C11—C13	114.8 (2)	C29—C30—H30	119.4
C7—C11—C12	108.1 (2)	C31—C30—H30	119.4
C13—C11—C12	110.8 (2)	C30—C31—C26	117.9 (2)
C7—C11—H11	107.6	C30—C31—C35	121.0 (2)
C13—C11—H11	107.6	C26—C31—C35	121.0 (2)
C12—C11—H11	107.6	C34—C32—C27	109.5 (2)
C11—C12—H12A	109.5	C34—C32—C33	110.7 (2)
C11—C12—H12B	109.5	C27—C32—C33	113.0 (2)
H12A—C12—H12B	109.5	C34—C32—H32	107.8
C11—C12—H12C	109.5	C27—C32—H32	107.8
H12A—C12—H12C	109.5	C33—C32—H32	107.8
H12B—C12—H12C	109.5	C32—C33—H33A	109.5
C11—C13—H13A	109.5	C32—C33—H33B	109.5
C11—C13—H13B	109.5	H33A—C33—H33B	109.5
H13A—C13—H13B	109.5	C32—C33—H33C	109.5
C11—C13—H13C	109.5	H33A—C33—H33C	109.5
H13A—C13—H13C	109.5	H33B—C33—H33C	109.5
H13B—C13—H13C	109.5	C32—C34—H34A	109.5
C19—C14—C15	122.2 (2)	C32—C34—H34B	109.5
C19—C14—N2	119.4 (2)	H34A—C34—H34B	109.5
C15—C14—N2	118.4 (2)	C32—C34—H34C	109.5
C16—C15—C14	117.5 (2)	H34A—C34—H34C	109.5
C16—C15—C20	120.6 (2)	H34B—C34—H34C	109.5
C14—C15—C20	121.8 (2)	C31—C35—C36	112.8 (3)
C17—C16—C15	120.7 (3)	C31—C35—C37	110.3 (2)
C17—C16—H16	119.7	C36—C35—C37	111.2 (3)
C15—C16—H16	119.7	C31—C35—H35	107.4
C18—C17—C16	120.7 (2)	C36—C35—H35	107.4
C18—C17—H17	119.6	C37—C35—H35	107.4
C16—C17—H17	119.6	C35—C36—H36A	109.5
C17—C18—C19	121.6 (3)	C35—C36—H36B	109.5
C17—C18—H18	119.2	H36A—C36—H36B	109.5
C19—C18—H18	119.2	C35—C36—H36C	109.5
C14—C19—C18	117.2 (2)	H36A—C36—H36C	109.5
C14—C19—C23	122.6 (2)	H36B—C36—H36C	109.5
C18—C19—C23	120.1 (2)	C35—C37—H37A	109.5
C15—C20—C21	114.0 (2)	C35—C37—H37B	109.5
C15—C20—C22	110.4 (2)	H37A—C37—H37B	109.5
C21—C20—C22	109.7 (3)	C35—C37—H37C	109.5
C15—C20—H20	107.5	H37A—C37—H37C	109.5
C21—C20—H20	107.5	H37B—C37—H37C	109.5
			
C2—N1—C1—N2	−3.4 (4)	N2—C14—C19—C23	−3.2 (3)
C2—N1—C1—N3	179.0 (2)	C17—C18—C19—C14	−0.8 (4)
C14—N2—C1—N1	168.7 (2)	C17—C18—C19—C23	−177.5 (2)
C14—N2—C1—N3	−13.7 (3)	C16—C15—C20—C21	31.2 (4)
C26—N3—C1—N1	−15.4 (3)	C14—C15—C20—C21	−152.1 (3)
C26—N3—C1—N2	166.85 (19)	C16—C15—C20—C22	−92.9 (3)
C1—N1—C2—C7	−103.3 (3)	C14—C15—C20—C22	83.9 (3)
N1—C2—C3—C4	161.8 (2)	C14—C19—C23—C25	−101.8 (3)
C7—C2—C3—C4	−8.6 (3)	C18—C19—C23—C25	74.7 (3)
C2—C3—C4—C5	4.8 (3)	C14—C19—C23—C24	135.6 (2)
C3—C4—C5—C6	0.9 (4)	C18—C19—C23—C24	−47.9 (3)
C4—C5—C6—C7	−3.0 (4)	C1—N3—C26—C27	88.2 (3)
N1—C2—C7—C6	−164.0 (2)	C1—N3—C26—C31	−94.8 (3)
C5—C6—C7—C2	−0.8 (3)	C31—C26—C27—C28	0.9 (3)
C4—C3—C8—C10	40.7 (3)	N3—C26—C27—C28	177.9 (2)
C2—C3—C8—C10	−148.0 (2)	C31—C26—C27—C32	−176.3 (2)
C4—C3—C8—C9	−83.4 (3)	N3—C26—C27—C32	0.7 (3)
C2—C3—C8—C9	87.9 (3)	C26—C27—C28—C29	−0.9 (4)
C2—C7—C11—C13	152.1 (2)	C32—C27—C28—C29	176.3 (2)
C6—C7—C11—C13	−37.5 (3)	C27—C28—C29—C30	0.4 (4)
C2—C7—C11—C12	−83.7 (3)	C28—C29—C30—C31	0.1 (4)
C6—C7—C11—C12	86.8 (3)	C29—C30—C31—C26	−0.1 (4)
C1—N2—C14—C19	−96.6 (3)	C29—C30—C31—C35	−177.7 (2)
C1—N2—C14—C15	84.0 (3)	C27—C26—C31—C30	−0.5 (3)
C19—C14—C15—C16	1.3 (3)	N3—C26—C31—C30	−177.4 (2)
N2—C14—C15—C16	−179.4 (2)	C27—C26—C31—C35	177.2 (2)
C19—C14—C15—C20	−175.6 (2)	N3—C26—C31—C35	0.2 (3)
N2—C14—C15—C20	3.8 (3)	C28—C27—C32—C34	−92.4 (3)
C14—C15—C16—C17	−1.0 (4)	C26—C27—C32—C34	84.7 (3)
C20—C15—C16—C17	175.9 (2)	C28—C27—C32—C33	31.5 (3)
C15—C16—C17—C18	−0.1 (4)	C26—C27—C32—C33	−151.4 (2)
C16—C17—C18—C19	1.1 (4)	C30—C31—C35—C36	−34.8 (4)
C15—C14—C19—C18	−0.4 (3)	C26—C31—C35—C36	147.6 (3)
N2—C14—C19—C18	−179.8 (2)	C30—C31—C35—C37	90.2 (3)
C15—C14—C19—C23	176.2 (2)	C26—C31—C35—C37	−87.3 (3)

### Table 1 Comparison of interatomic distances (Å, °) of (I) with free guanidine, (II)

**Table:**

	C1—N1	C1—N2	C1—N3	N1—C1—N2	N2—C1—N3	N3—C1—N1
(I)	1.287 (3)	1.361 (3)	1.374 (3)	125.0 (2)	115.57 (19)	119.42 (19)
(II)	1.316 (2)	1.348 (2)	1.357 (2)	121.99 (13)	118.47 (14)	119.52 (13)
